# Use of N-(l-naphthyl)-ethylendiamine-dihydrochloride (NEDD) as secondary calibrator for conjugated bilirubin on the DuPont aca

**DOI:** 10.1155/S1463924685000098

**Published:** 1985

**Authors:** Maria Luisa Gozzo, Giuliano Barbaresi, Francesco Dalla Torre, Gianpaolo Littarru, Cecilia Zuppi

**Affiliations:** Istituto di Fisiologia Umana Laboratorio di Chimica Clinica Policlinico Universitario A. Gemelli Largo A. Gemelli 8 Roma 00168 Italy


Journal of Automatic Chemistry, Volume 7, Number 1 (January–March 1985), pages 43–45

### Use of N-(1-naphthyl)-ethylendiamine-dihydrochloride (NEDD) as secondary calibrator for conjugated bilirubin on the DuPont aca

**Maria Luisa Gozzo, Giuliano Barbaresi, Francesco Dalla Torre, Gianpaolo Littarru and Cecilia Zuppi**

*Istituto di Fisiologia Umana, Laboratorio di Chimica Clinica, Policlinico Universitario A. Gemelli, Largo A. Gemelli 8, 00168 Roma, Italy*

### Introduction

The calibration of analytical methods for conjugated bilirubin (CBil) has some well-known difficulties, all of which are due to the lack of stability of the bilirubin glycuronides.

N-(1-naphthyl)-ethylendiamine-dihydrochloride (NEDD) and its derivatives have been used as secondary calibrators for continuous-flow analytical instruments (for example Technicon's SMAC) [1]. These molecules are known to react with the diazonium salts, yielding azoderivatives which have spectral qualities similar to those of azobilirubin. NEDD has also successfully been employed in the authors' laboratory as secondary calibrator for conjugated bilirubin on a discrete clinical analyser: the Hitachi 706 [2].

Results obtained for a calibration procedure based on the use of NEDD, for the CBil method on DuPont's Automatic Clinical Analyser (aca), are reported here. In the aca method for CBil determination [3], quantitative measurement of conjugated bilirubin in serum is accomplished by the reaction of the conjugated bilirubin with p-nitro-benzen-diazonium tetrafluoroborate (PNB) under acid conditions, to yield a red chromophore, the absorbance of which is measured by a two wavelength end-point technique.

Since no adequate CBil standard is available, DuPont recommends that this method be calibrated using a secondary TBil calibrator (or a TBil standard) to which an 'apparent CBil' value is assigned. The entire procedure consists of three steps:

(1) Initial calibration of the CBil channel by correlation to a reference method using patient samples.
(2) Pretreatment of CBil packs with Tween 20, followed by analysis of a TBil calibrator to obtain an 'apparent CBil' value.
(3) Calibration of new lots of CBil aca packs.

For a new lot of TBil calibrator a new 'apparent CBil' value should be found.

An alternative procedure, proposed here, consists of calibrating the aca CBil channel using NEDD as secondary calibration material to which CBil values were assigned using the DuPont-certified TBil calibrator.

### Materials and methods

### *Instruments*

(1) Automatic clinical analyser: aca II (DuPont Instruments, Wilmington, Delaware 19898, USA).
(2) Spectrophotometer: Uvicon 810 (Kontron Ltd Analytic International, Bernerstrasse Sud 169, CH 8048 Zurich, Switzerland).

### *Reagents*

DuPont Total Bilirubin Calibrator lot No. 2BD615 (DuPont Company) and N-(1-naphthyl)-ethylendiamine-dihydrochloride (NEDD) (Fluka AG-Buchs SG, Switzerland) were used for calibration. Analytical test packs for CBil (DuPont) (beared lot No. P2861 A). All other chemical products were of the purest grade commercially available. Deionized distilled-water was used to prepare all reagents.

### *Methods*

The method of Malloy and Evelyn [4] was used as a reference manual method for total and conjugated bilirubin determination. The reference method was calibrated by means of the standard DuPont TBil calibrator lot No. 2BD615 (target value = 204 mg/l). Even though this is not an NBS-certified material, it is DuPont's officially recommended calibrator for the aca CBil method; the authors considered it convenient to have calibrations for TBil and CBil originating from the same material.

### *Proposed aca calibration procedure*

Linear relation between concentration and optical density was obtained for the TBil calibrator processed by the manual Malloy-Evelyn procedure for TBil (see figure 1[*a*]). An analogous linear relation was determined for NEDD, again using the manual Malloy-Evelyn procedure for CBil (figure 1 [*b*]). The ratio of the two obtained slopes represents the conversion factor used for assigning the 'apparent CBil' value to NEDD. NEDD solutions were then used as reference material for calibrating the aca's CBil channel according to the general procedure proposed by DuPont for substrates [3].

The rationale of this procedure lies in the fact that molar absorptivities of the diazo products for conjugated and total bilirubin are commonly assumed to be the same [4].

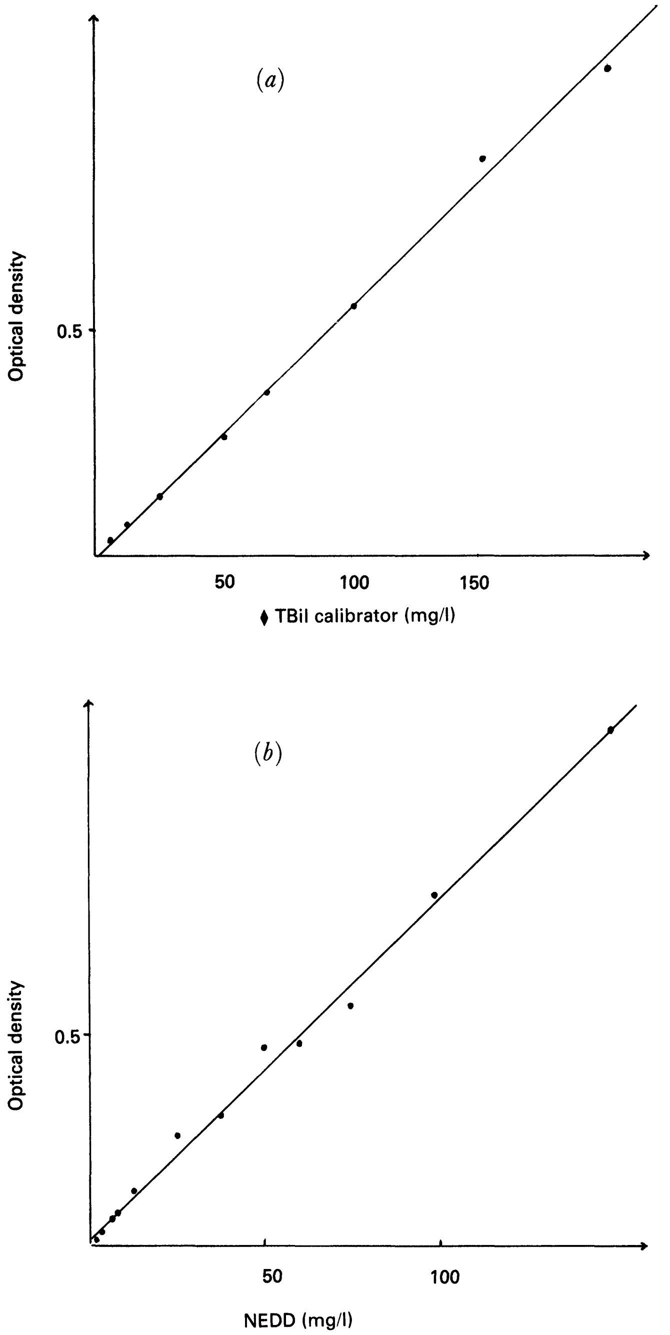

*Figure 1. Linear relations between concentrations as mg/l (x-axis) and optical densities (y-axis):*
*(a) Total bilirubin:* $y = -0{\cdot}0057 + 0{\cdot}0055 \times$
$S_{y_x} = 0{\cdot}024 \quad r = 0{\cdot}9983$
*(b) NEDD:* $y = 0{\cdot}0149 + 0{\cdot}0075 \times$
$S_{y_x} = 0{\cdot}027 \quad r = 0{\cdot}9973.$
*TBil concentrations were obtained by serial dilutions of DuPont TBil calibrator with deionized distilled-water. NEDD concentrations were obtained by dilution of an aqueous stock solution of 500 mg/l. All observed absorbances were the mean of three determinations.*

The problem that the molar absorptivity of the azo-NEDD product is different from that of azo-bilirubin product can be overcome by establishing the conversion factor, which allows the 'apparent CBil' value to be assigned to NEDD solutions.

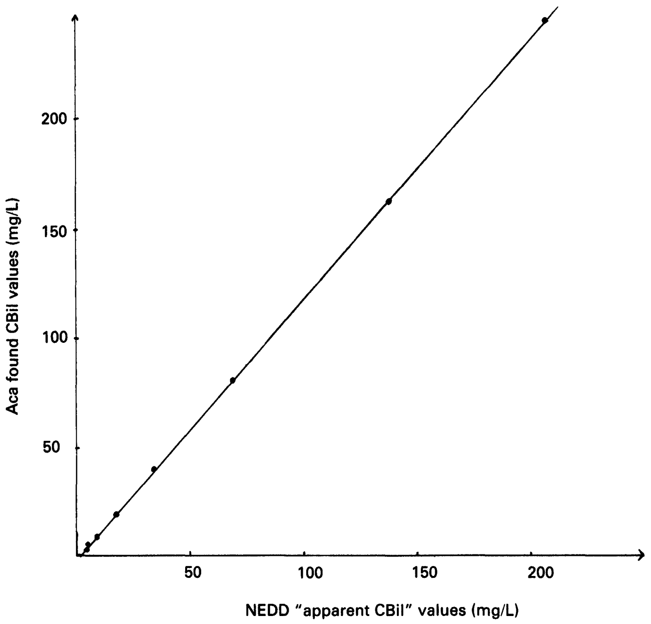

*Figure 2. Aca calibration plot before setting adjustment: NEDD 'apparent CBil' values (x-axis) versus aca found values (y-axis).*
$Y = -0{\cdot}1838 + 1{\cdot}1978\,x$
$S_{y_x} = 0.050 \quad r = 1{\cdot}000.$
*Found aca values are the mean of three different CBil packs according to the general DuPont calibration procedure.*

### Results and discussion

The conversion factor found for NEDD was 1·38 (1 mg NEDD = 1·38 mg CBil). Lot-to-lot variations of TBil calibrator, as well as new preparations of NEDD solutions, or use of a NEDD with different purity, might reasonably be assumed to result in different conversion factors. Bilissis *et al.* [5] refer a conversion factor of 1·0. The authors' experience is that conversion factor values (for the past three years) were between 1·38 and 1·48. It should be noted that 'actual' conversion factor and 'apparent CBil' values are always referred to the DuPont-certified TBil calibrator. The main concern was to obtain a dependable method for lot-to-lot recalibration of CBil packs, rather than improving accuracy by using a primary calibrator.

Figure 2 shows the aca calibration plot of expected CBil values (NEDD 'apparent CBil' values) versus aca found values. This graph was, of course, obtained with the instrument calibrated according to the standard procedure suggested by DuPont [3]. Subsequent adjustment of starting point and scale factor was then performed according to this correlation. The extent of settings variation needed, was quite small, but this was not surprising since the DuPont suggested aca recalibration methodology also refers, though a rather involved procedure, to the same calibration material.

In order to evaluate the validity of the proposed calibration procedure, a split sample comparison between manual reference method and aca was performed. The related plot (figure 3) clearly indicates a flexus point around CBil values of 50 mg/l. On the basis of this pattern, two more regression lines for values below and over 50 mg/l respectively were traced: intercepts and

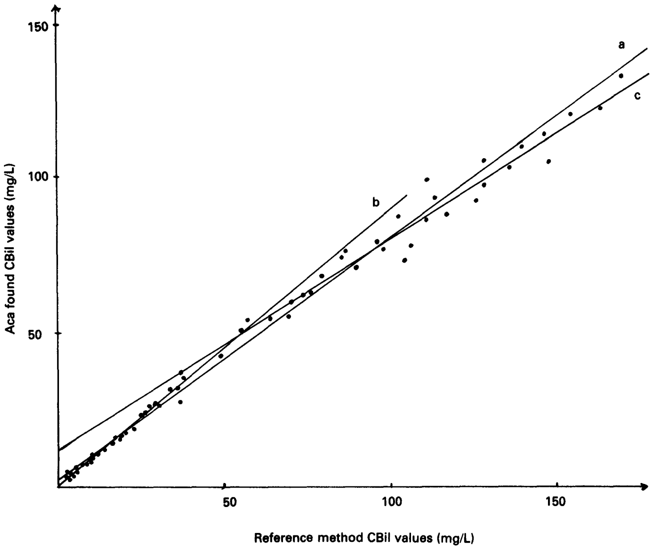

*Figure 3. Plot of the split sample comparison between Malloy-Evelyn reference method and aca:*
*(a) This line represents the correlation for CBil values from 1 mg/l up to 170 mg/l:* $N = 60\ y = 2{\cdot}3127 + 0{\cdot}7810\ x$
$S_{y_x} = 4{\cdot}004 \quad r = 0{\cdot}9930$
*(b) This line represents the correlation for CBil values below 50 mg/l:* $y = 0{\cdot}4642 + 0{\cdot}8872\ x$
$S_{y_x} = 1{\cdot}634 \quad r = 0{\cdot}9905$
*(c) This line represents the correlation for CBil values above 50 mg/l:* $y = 11{\cdot}033 + 0{\cdot}6943\ x$
$S_{y_x} = 5{\cdot}678 \quad r = 0{\cdot}9964.$

slopes are quite different for the two levels. This fact was not unexpected. A previous report from Bovie *et al.* [6] points out the unacceptable bias between DuPont aca and SMAC (Jendrassik and Grof) for CBil levels below 20 mg/l. The authors propose the use of two different aca channels: one properly calibrated for CBil values above 20 mg/l and the other one for values below 20 mg/l.

The calibration procedure proposed behaves satisfactorily for CBil values up to 50 mg/l, as confirmed by Total Error (TE) [7] referring to values in this range. In fact, at 10 mg/l level TE was 1·61 mg/l with the lower limit ($TE_l$) = 1·46 mg/l and the upper limit ($TE_u$) = 1·84 mg/l. This is quite acceptable: a medical significance value of 2 mg/l has been proposed by Barnett [8].

Systematic underestimation of CBil values above 50 mg/l can be overcome by dilution. This may represent a reasonable solution which avoids dedicating two aca channels to CBil determinations. The proposed calibration of the aca CBil method has been used by the authors for the past three years. NEDD solutions showed a long-term stability (more than 12 months) when kept in the dark at 0–4 °C. However, new solutions were made and new 'apparent CBil' values were assigned to NEDD once a year. Lot changes in aca packs supplied in this period only require aca recalibration against NEDD solutions.

In conclusion, this simple, reliable procedure for calibrating the aca's CBil channel is a good alternative to the rather involved official methodology.

### References

1. Furda, J., Morgenstern, S. and Snyder, L. R., *Clinical Chemistry,* **22** (1976), 1042.
2. Giocoli, G., Gozzo, M. L., Miggiano, G. and e Martorana, G. E., *Laboratory Journal of Research and Laboratory Medicine,* **9** (1982), 469.
3. *Aca Chemistry Instruction Manual PN 703492-901* (DuPont Company Clinical System Division, Wilmington, Delaware 19898, USA).
4. Determination of direct and total bilirubin by the diazo reaction. In *Clinical Chemistry Principles and Tecnics,* edited by Henry, R. J., Connon, D. C. and Winkelman, J. W. (Harper & Row, London, 1974), 1051.
5. Bilissis, P. K. and Speer, R. J., *Clinical Chemistry,* **9** (1963), 552.
6. Bovie, L. J., Duhal, J. C. and Kirkpatrick, P. B. *Clinical Chemistry,* **28** (1982), 385.
7. Westgard, J. O., Carey, R. N. and Wold, S. *Clinical Chemistry,* **20** (1974), 825.
8. Barnett, R. N., *American Journal of Clinical Pathology,* **50** (1968), 671.

### COLLOQUIUM SPECTROSCOPIUM INTERNATIONALE XXIV

*To be held from 15 to 21 September 1985 in Garmisch-Partenkirchen, FR Germany*

CSI XXIV will touch upon all areas of analytical chemistry: an international group of spectroscopists is expected to attend. Programme topics include:

*Basic theory and methods*—
Atomic emission spectroscopy, Atomic absorption spectrometry, Atomic fluorescence spectrometry, Infra-red and Raman spectroscopy, Molecular spectroscopy (UV and Vis), Laser spectroscopy, Mass spectroscopy (organic and inorganic), Standard reference materials etc.

*Analytical applications for specific problems*—
Analysis of metals, Analysis of different industrial products, Geochemical analysis, Biological, clinical and pharmaceutical analysis, Analysis in agriculture and nutrition chemistry, Environmental analysis.

*The second circular will soon be available from CSI XXIV, Organisationsbüro, Institut für Spektrochemie und angewandte Spektroskopie, Postfach 778, D 4600 Dortmund 1, FR Germany.*

